# Proanthocyanidins Ameliorate LPS-Inhibited Osteogenesis of PDLSCs by Restoring Lysine Lactylation

**DOI:** 10.3390/ijms25052947

**Published:** 2024-03-03

**Authors:** Yaxin Wu, Xiangyao Wang, Yuxiao Zhang, Zhihao Wen, Yuanyuan Li, Kehan Zhang, Nuerlan Gosar, Qilin Li, Jing Mao, Shiqiang Gong

**Affiliations:** 1 Department of Stomatology, Tongji Hospital, Tongji Medical College, Huazhong University of Science and Technology, Wuhan 430030, China; yaxinwu@163.com (Y.W.); d202380929@hust.edu.cn (X.W.); m202372648@hust.edu.cn (Y.Z.); kqys_wzh@163.com (Z.W.); lyy13486378721@163.com (Y.L.); 15987879508@163.com (K.Z.); gaosaer0606@163.com (N.G.); qilinli@tjh.tjmu.edu.cn (Q.L.); 2 School of Stomatology, Tongji Medical College, Huazhong University of Science and Technology, Wuhan 430033, China

**Keywords:** periodontitis, lysine, proanthocyanidins, bone regeneration

## Abstract

Periodontitis is a bacteria-induced inflammatory disease characterized by the progressive destruction of periodontal supporting tissues. Periodontal ligament stem cells (PDLSCs) are capable of differentiating into osteoblasts, which is an important stem cell source for endogenous periodontal tissue regeneration. Lysine lactylation (Kla) is a novel post-translational modification of proteins that is recently thought to be associated with osteogenic differentiation. Here, we found that lactylation levels are reduced both in the periodontal tissue of rats with periodontitis and lipopolysaccharide (LPS)-stimulated human PDLSCs. Proanthocyanidins were able to promote the osteogenesis of inflamed PDLSCs by restoring lactylation levels. Mechanistically, proanthocyanidins increased lactate production and restored the lactylation levels of PDLSCs, which recovered osteogenesis of inflamed PDLSCs via the Wnt/β-catenin pathway. These results provide evidence on how epigenetic regulation by pharmacological agents influence the osteogenic phenotype of stem cells and the process of periodontal tissue repair. Our current study highlights the valuable potential of natural product proanthocyanidins in the regenerative engineering of periodontal tissues.

## 1. Introduction

Periodontitis, an inflammatory disease initiated by a bacterial pathogen, is the most common oral disease and accounts for a considerable global public health burden [1,2]. It is characterized by the progressive destruction of the periodontal supporting tissues, especially the loss of the alveolar bone [3]. With the influence of pathogenic microorganisms, the balance between bone resorption and bone regeneration in the alveolar bone is disrupted, as evidenced by the activation of osteoclasts and the inhibition of osteoblasts, leading to alveolar bone loss and, in severe cases, tooth loss [4]. Current treatments for periodontitis include the removal of local irritants, infection control, and the promotion of periodontal tissue repair [5].

Periodontal ligament stem cells (PDLSCs), oral-derived mesenchymal stem cells, were first identified in human periodontal tissues in 2004 [6]. PDLSCs have the potential to differentiate into a variety of cell types, including osteoblasts, odontogenic osteocytes, chondroblasts, and adipocytes [7]. In vivo studies have shown that PDLSCs are capable of forming the alveolar bone, periodontal membrane, and osteoid-like tissues to repair periodontal defects [8]. PDLSCs also have a strong clonal self-renewal ability, and human PDLSCs show higher growth potential than human bone marrow-derived MSCs and human dental pulp stem cells [9], which makes PDLSCs a promising candidate for repairing the periodontal defect. In recent years, several studies have found that the osteogenic differentiation of PDLSCs is significantly weakened in the inflammatory environment induced by lipopolysaccharide (LPS) or tumor necrosis factor-α (TNF-α) [10,11], which directly impairs the reparative role of PDLSCs in the local inflammatory environment of periodontitis. Therefore, how to restore the osteogenic differentiation of PDLSCs in the inflammatory environment and then recover the structure and function of the defective alveolar bone tissues is essential for periodontal tissue regeneration therapy based on stem cell technology.

Lactate, a major product of cellular glycolysis, was once regarded as a “metabolic waste product” [12]. As the exploration of lactate continues, accumulating evidence has suggested the crucial roles of lactate in bone homeostasis and bone repair. Secreted by many different pro-inflammatory cells in response to aerobic/anaerobic glycolysis, lactate can exert multiple functions in the early stages of bone defect repair [13]. For example, lactate has been reported to induce angiogenic responses in endothelial cells by up-regulating the expression of angiogenic factors (e.g., vascular endothelial growth factor) and activating the pro-angiogenic nuclear factor-κB-IL-8 pathway [14]. Lactate secreted into the extracellular matrix could also recruit mesenchymal stem cells to the local bone microenvironment [15]. In addition, lactate stimulates collagen deposition in an autocrine and paracrine manner, thereby contributing to extracellular matrix synthesis, cartilage scab progression, and bone regeneration [16]. Lysine lactylation (Kla) is a novel post-translational modification of proteins, first reported in 2019, which demonstrated that lactate accumulated during metabolism can act as a precursor substance leading to lactylation modification of lysine residues [17]. Lactylation includes both histone modifications and non-histone modifications, which are engaged in tumor cell proliferation [18], nervous system regulation [19], metabolic regulation [20], and other important life activities. The discovery of lactylation has provided new thoughts on the mechanism of lactate’s role in osteogenic differentiation. Recently, it has been found that endothelial cell glycolysis promotes the differentiation of bone marrow mesenchymal stem cells to osteoblasts through histone lactylation [21]. However, the role of lactylation in the osteogenic differentiation of PDLSCs in periodontitis remains unclear.

Proanthocyanidins (PA) are a class of polyphenolic compounds found widely in the plant kingdom and are natural antioxidants in plants [22]. Proanthocyanidins have been reported to be able to influence the osteoclastic and osteoblastic processes of bone-related cells. Grape seed proanthocyanidin extract can prevent bone loss by regulating osteoclast differentiation, apoptosis, and proliferation [23]. Proanthocyanidins can promote osteogenic differentiation of human periodontal ligament cells in inflammatory environments by inhibiting the NF-κB signaling pathway [24]. Moreover, our recent study found that proanthocyanidins activate the autophagy of PDLSCs by inhibiting the PI3K/Akt/mTOR signaling pathway, thereby promoting osteogenesis and enhancing endogenous alveolar bone regeneration [25]. However, the specific mechanism by which proanthocyanidins promote the osteogenic differentiation of PDLSCs in the inflammatory state remains to be refined. 

Given the important role of lactylation in the osteogenic differentiation in osteoprogenitor cells, the aim of this study was to investigate whether proanthocyanidins could enhance the osteogenic capacity of LPS-stimulated PDLSCs by restoring lactylation. In the inflammatory state of PDLSCs induced by LPS, we landed on lactate production, which is closely related to lactylation, and found that proanthocyanidins were able to enhance the osteogenesis of PDLSCs by restoring lactylation through increasing lactate production and further analyzed the underlying mechanism.

## 2. Results

### 2.1. Osteogenesis and Lactylation Levels Are Decreased in the Periodontal Tissue of Periodontitis Rats and LPS-Stimulated Human PDLSCs

**Figure 1:**
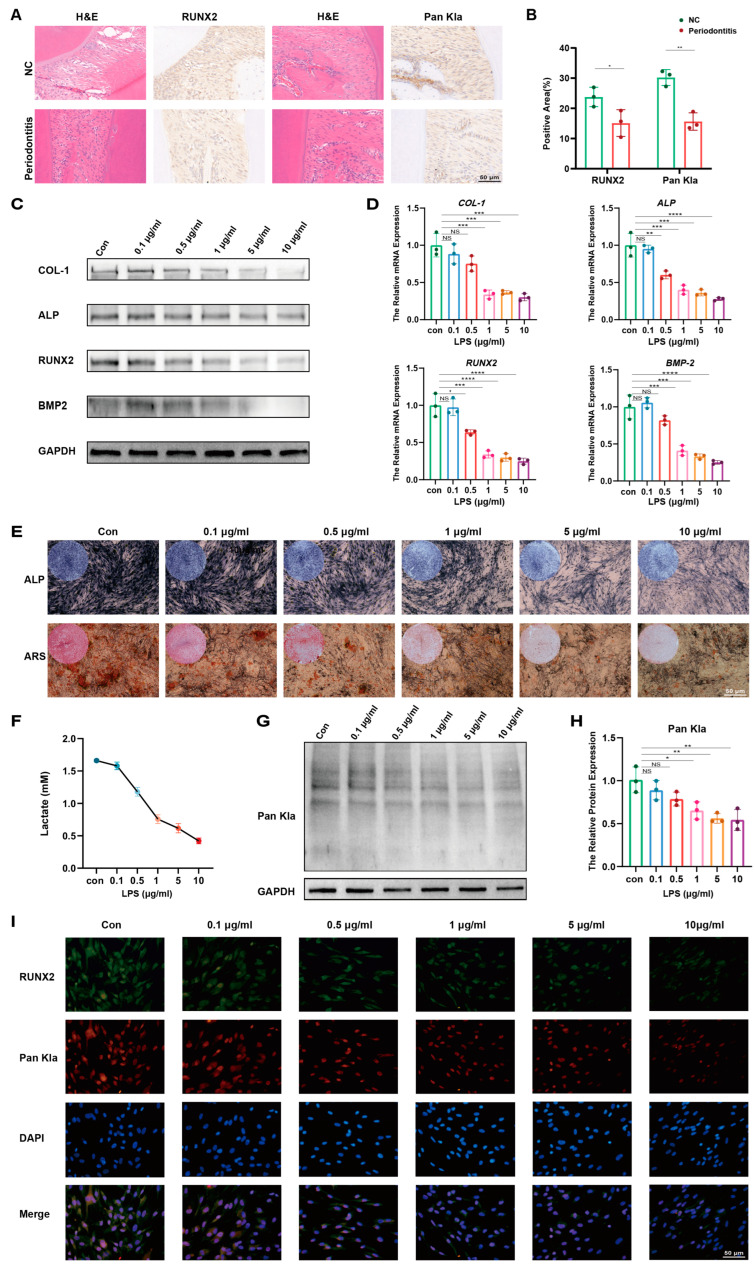
Osteogenesis and lactylation levels are decreased in inflamed periodontal tissue and LPS-stimulated PDLSCs. (**A**) H&E staining in rat periodontal tissues and immunohistochemical Pan Kla and RUNX2 expression. (**B**) Quantitative analysis of positive areas. (**C**) Western blotting showing protein levels of COL-1, ALP, RUNX2, and BMP2 in LPS-treated PDLSCs. (**D**) qRT-PCR showing mRNA levels of osteogenic genes *COL-1*, ALP, *RUNX2*, and *BMP2*. (**E**) ALP staining at 7 days and ARS staining at 21 days of osteogenic induction in LPS-treated PDLSCs. (**F**) Lactate concentration in the culture medium of PDLSCs was measured at the indicated times, as shown in the quantitative analysis. (**G**) Western blotting showing the protein level of Pan Kla in LPS-treated PDLSCs. (**H**) Quantitative analysis of Pan Kla protein levels. (**I**) Immunofluorescence staining images showing the expression levels of RUNX2 (green) and Pan Kla (red) in PDLSCs after LPS treatment. Cell nuclei were stained with DAPI (blue). PDLSCs: periodontal ligament stem cells; Kla: lysine lactylation; LPS: lipopolysaccharide; NC: healthy mice without periodontitis; H&E: hematoxylin and eosin staining (Data are expressed as mean ± SD, N = 3. * *p* < 0.05, ** *p* < 0.01, *** *p* < 0.001, **** *p* < 0.0001, NS indicates no statistical difference).

H&E staining of sample sections showed significant inflammatory cell infiltration in the periodontitis group. Immunohistochemical staining showed that both osteogenic-associated RUNX2 and Pan Kla were decreased in the periodontitis group compared to the control group (Figure 1A). The difference in the area of positive areas for both RUNX2 and Pan Kla between the NC and periodontitis groups was statistically significant (Figure 1B). Then, *P. gingivalis*-derived LPS was used to construct an inflammation model of PDLSCs (Figure S1), and we first explored the effect of LPS on the osteogenic differentiation of PDLSCs. Western blot results showed that with the increase in LPS-stimulated concentration, the expression of PDLSC osteogenic-associated proteins COL-1, ALP, RUNX2, and BMP2 was gradually decreased (Figure 1C). Consistent with the protein level, qRT-PCR results showed that the levels of osteogenesis-related genes in PDLSCs also showed a concentration-dependent decrease after LPS treatment compared with the control group (Figure 1D). To rigorously test the effect of LPS on the osteogenic directed differentiation ability of PDLSCs, we used an osteogenic induction medium to induce directed osteogenic differentiation of PDLSCs, and the results of ALP (7 d) and ARS (21 d) staining showed a gradual decrease in alkaline phosphatase and calcium nodule production in PDLSCs with the increase in LPS concentration compared with the control group (Figure 1E). Since changes in lactate production have been shown to be closely associated with the development of a variety of diseases, we examined the changes in lactate production in PDLSCs in the inflammatory state. The results of the lactate concentration assay experiments showed a significant decrease in lactate concentration in the supernatants of PDLSCs after LPS treatment (Figure 1F). We then examined the changes in lactylation levels after LPS treatment, and interestingly, the changes in lactate production and lactylation levels in PDLSCs after LPS treatment were strikingly consistent with their alterations in osteogenic differentiation-associated proteins, especially RUNX2 (Figure 1G,H). We detected the distribution of Pan Kla and osteogenesis-associated RUNX2 proteins in cells using specific antibodies, respectively, and observed that the immunofluorescence signals of both Pan Kla and RUNX2 were gradually weakened after LPS treatment, and the co-localization signals of the two showed a gradual decrease (Figure 1I). In summary, the osteogenic differentiation of PDLSCs was significantly reduced in the LPS-induced inflammatory state, which was accompanied by a decrease in lactate production and lactylation levels. Therefore, we deduced that there was a correlation between lactylation and the osteogenic differentiation of PDLSCs.

### 2.2. Restoration of Lactylation by Trichostatin A Recovers the Osteogenesis of PDLSCs in the Inflammatory State

**Figure 2:**
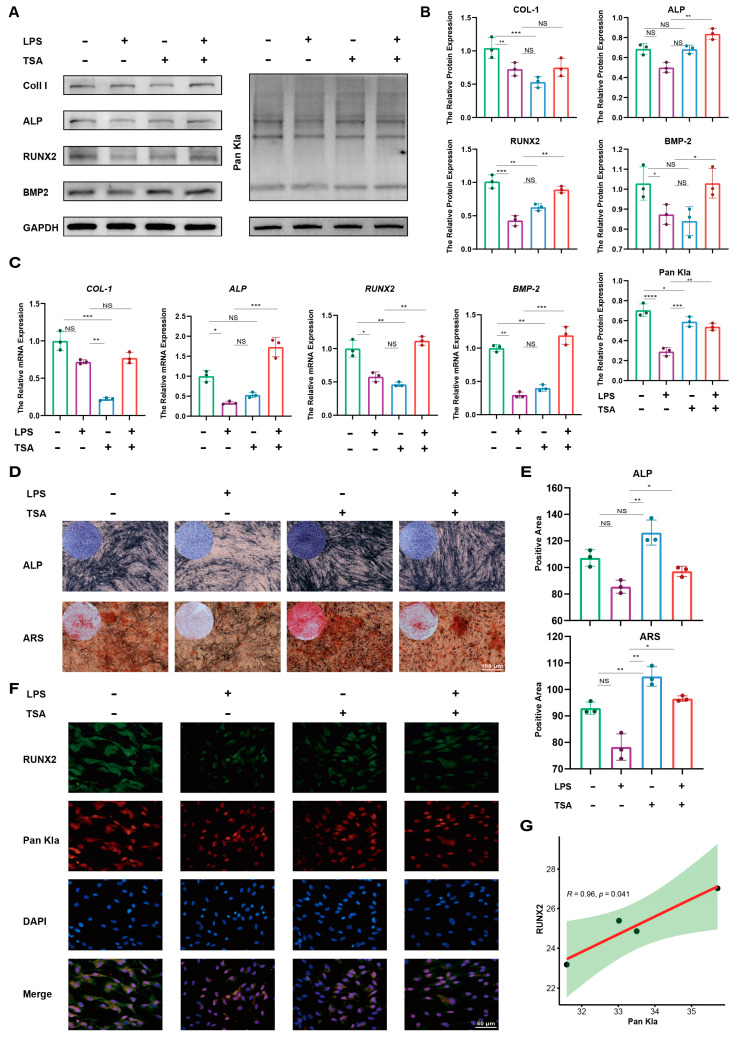
Restoration of lactylation by TSA recovers the osteogenesis of PDLSCs in an inflammatory state. (**A**) Western blotting showing the protein levels of COL-1, ALP, RUNX2, and BMP2 in PDLSCs after treatment with the conditions as shown. (**B**) Quantification of protein levels of COL-1, ALP, RUNX2, and BMP2. (**C**) qRT-PCR showing mRNA levels of osteogenic genes *COL-1*, ALP, *RUNX2,* and *BMP2*. (**D**) ALP staining at 7 days and ARS staining at 21 days of osteogenic induction in PDLSCs. (**E**) Quantitative analysis of ALP staining and ARS staining. (**F**) Immunofluorescence staining images showing the expression levels of RUNX2 (green) and Pan Kla (red) in PDLSCs treated with the conditions as shown. Cell nuclei were stained with DAPI (blue). (**G**) Pearson linear regression analysis of RUNX2 and Pan Ka fluorescence intensity. PDLSCs: periodontal ligament stem cells; Kla: lysine lactylation; LPS: lipopolysaccharide; TSA: Trichostatin A (Data are expressed as mean ± SD, N = 3. * *p* < 0.05, ** *p* < 0.01, *** *p* < 0.001, **** *p* < 0.0001, NS indicates no statistical difference).

Since lactylation in PDLSCs is down-regulated in the inflammatory state, we wondered whether restoring lactylation levels in PDLSCs in the inflammatory state could recover their osteogenic differentiation. In light of this, we performed an osteogenic induction of PDLSCs in the presence of LPS. The lactylation eraser inhibitor Trichostatin A (TSA) was applied to detect the effect of lactylation restoration on the osteogenic differentiation of PDLSCs in the inflammatory state. Based on the results of previous experiments, we selected 10 μg/mL as the LPS stimulation concentration in the PDLSC inflammation model. Western blot results showed that compared with the control group, LPS stimulation resulted in a weakening of the expression of proteins related to the osteogenic differentiation of PDLSCs, accompanied by a significant decrease in lactylation levels. The treatment of TSA was able to increase the level of lactylation and significantly restore the expression of ALP, RUNX2, and BMP2 (Figure 2A,B), and the qRT-PCR results showed that TSA was able to partially restore the osteogenic differentiation genes ALP and BMP2 in PDLSCs that were reduced by LPS (Figure 2C). Similarly, PDLSCs in the TSA-only treatment group formed more alkaline phosphatase and mineralized nodules after 7 d and 21 d of osteogenic induction compared with the LPS-treated group, and the addition of TSA partially rescued the LPS-inhibited osteogenic capacity of PDLSCs (Figure 2D,E). Immunofluorescence results showed that TSA restored the fluorescence intensity of Pan Kla and RUNX2 in the LPS-treated group (Figure 2F), and there was a correlation between the fluorescence intensities of RUNX2 and Pan Kla (Figure 2G). These results suggest that the decreased osteogenic differentiation ability of LPS-treated PDLSCs may be caused by decreased lactylation, and restoring the lactylation level of PDLSCs in the inflammatory state can restore their osteogenic differentiation.

### 2.3. Proanthocyanidins Promote Osteogenesis and Elevate Lactylation of PDLSCs

**Figure 3:**
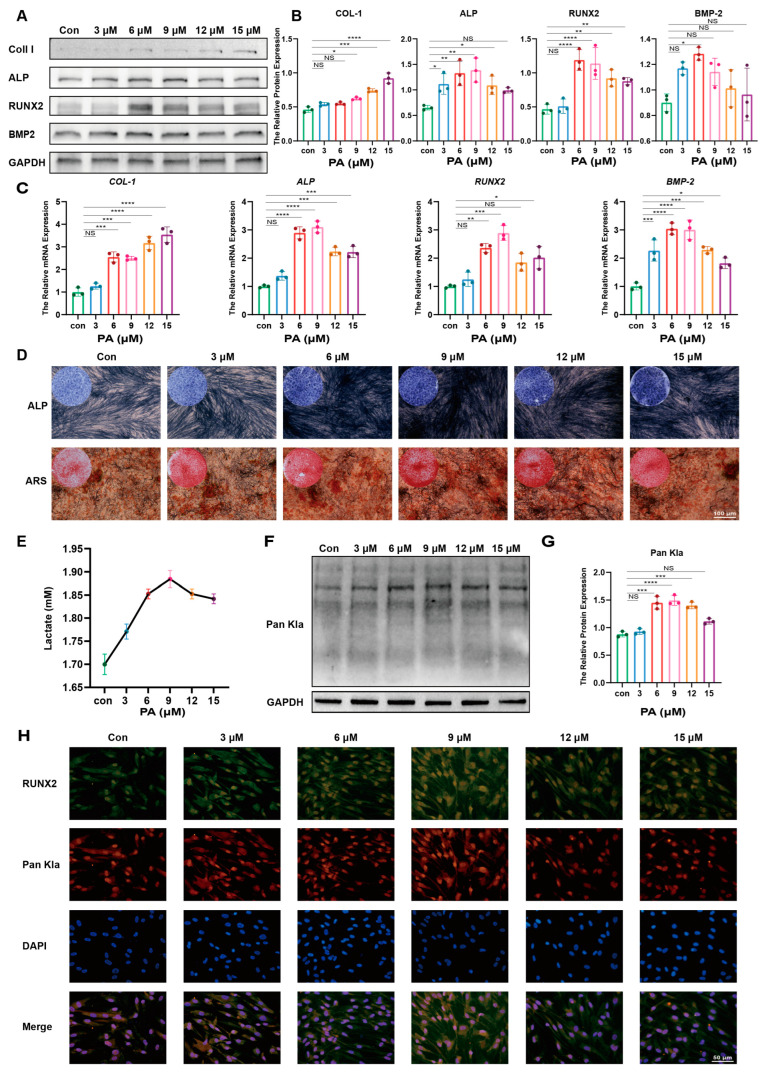
Proanthocyanidins promote osteogenesis and elevate the lactylation of PDLSCs. (**A**) Western blotting showing protein levels of COL-1, ALP, RUNX2, and BMP2 in PA-treated PDLSCs. (**B**) Quantification of protein levels of COL-1, ALP, RUNX2, and BMP2. (**C**) qRT-PCR showing mRNA levels of osteogenic genes *COL-1*, ALP, *RUNX2*, and *BMP2*. (**D**) ALP staining at 7 days and ARS staining at 21 days of osteogenic induction in PA-treated PDLSCs. (**E**) Lactate concentration in the culture medium of PDLSCs was measured at the indicated times as shown in the quantitative analysis. (**F**) Western blotting showing the protein level of Pan Kla in PA-treated PDLSCs. (**G**) Quantitative analysis of Pan Kla protein levels. (**H**) Immunofluorescence staining images showing the expression levels of RUNX2 (green) and Pan Kla (red) in PDLSCs after treatment with the conditions as shown. Cell nuclei were stained with DAPI (blue). PDLSCs: periodontal ligament stem cells; Kla: lysine lactylation; PA: proanthocyanidins (Data are expressed as mean ± SD, N = 3. * *p* < 0.05, ** *p* < 0.01, *** *p* < 0.001, **** *p* < 0.0001, NS indicates no statistical difference).

Proanthocyanidins have been reported to promote osteogenic differentiation of PDLSCs in vitro and affect acetylation modification. In order to investigate whether proanthocyanidins affect the level of lactylation in PDLSCs, we added gradient concentrations of proanthocyanidins to the osteogenic induction medium. The results of the western blot experiments showed that proanthocyanidin treatments not only increased PDLSC osteogenic differentiation-related COL-1, ALP, RUNX2, and BMP2 protein expression (Figure 3A,B) but also increased the level of PDLSC osteogenic differentiation-related genes (Figure 3C). Meanwhile, we examined the effect of proanthocyanidins on the alkaline phosphatase and mineralized nodule formation capacity of PDLSCs and found that at a concentration of 9 μM, proanthocyanidins significantly promoted alkaline phosphatase and mineralized nodule formation in PDLSCs (Figure 3D). We subsequently examined the effect of proanthocyanidins on lactate production in PDLSCs and found that the lactate concentration in the supernatants of PDLSCs was significantly higher after proanthocyanidin treatment compared with the control group (Figure 3E) and that 9 μM still had the most significant effect. Therefore, we chose 9 μM as the stimulating concentration of proanthocyanidins for the subsequent experiments. We then detected the changes in the lactylation level of PDLSCs after proanthocyanidin treatment, and as expected, the lactate production of PDLSCs increased after proanthocyanidin treatment (Figure 3F), and there was a consistency in the alterations of lactate production, lactylation level, and osteogenic differentiation protein RUNX2 (Figure 3G). In addition, the results of immunofluorescence experiments showed a similar trend, i.e., proanthocyanidin treatment significantly elevated the fluorescence intensity of Pan Kla and RUNX2 in PDLSCs, and 9 μM was the peak fluorescence value (Figure 3H). In summary, the osteogenic differentiation of PDLSCs was significantly elevated by proanthocyanidin treatment and reached the maximum at 9 μM, accompanied by the elevation of lactate production and lactylation levels, and the trends of the three changes were similar, which aroused our thoughts on whether proanthocyanidins affect the osteogenic differentiation of PDLSCs by affecting the lactylation of PDLSCs.

### 2.4. Proanthocyanidins Promote PDLSC Osteogenesis by Restoring Lactylation of PDLSCs in Inflammatory States

**Figure 4:**
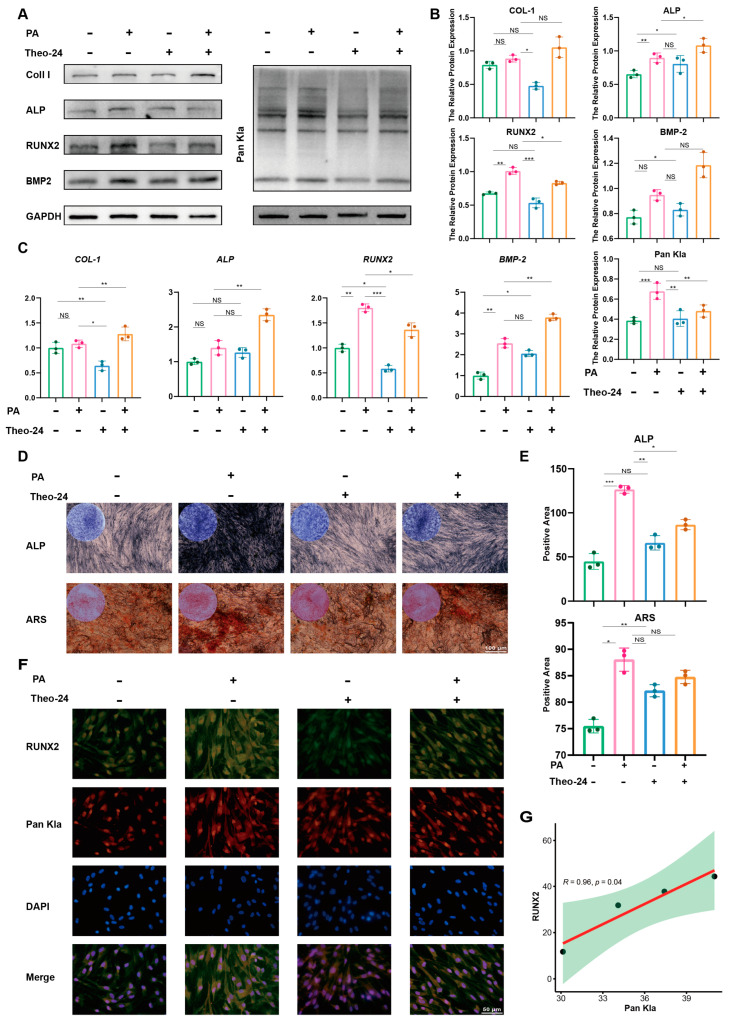
Proanthocyanidins recover PDLSC osteogenesis by restoring the lactylation of PDLSCs in an inflammatory state. (**A**) Western blotting showing the protein levels of COL-1, ALP, RUNX2, and BMP2 in PDLSCs after treatment with the conditions as shown. (**B**) Quantification of protein levels of COL-1, ALP, RUNX2, and BMP2. (**C**) qRT-PCR showing mRNA levels of osteogenic genes *COL-1*, ALP, *RUNX2,* and *BMP2*. (**D**) ALP staining at 7 days and ARS staining at 21 days of osteogenic induction in PDLSCs. (**E**) Quantitative analysis of ALP staining and ARS staining. (**F**) Immunofluorescence staining images showing the expression levels of RUNX2 (green) and Pan Kla (red) in PDLSCs treated with the conditions as shown. Cell nuclei were stained with DAPI (blue). (**G**) Pearson linear regression analysis of RUNX2 and Pan Ka fluorescence intensity. PDLSCs: periodontal ligament stem cells; Kla: lysine lactylation; PA: proanthocyanidins; Theo-24: theophylline (Data are expressed as mean ± SD, N = 3. * *p* < 0.05, ** *p* < 0.01, *** *p* < 0.001, NS indicates no statistical difference).

Given that both osteogenesis and lactylation levels were up-regulated in PDLSCs after proanthocyanidin treatment, we investigated whether the promoting effect of proanthocyanidins on the osteogenic differentiation of PDLSCs in the inflammatory state was dependent on lysine lactylation. Therefore, we induced an inflammatory state by LPS after the osteogenic induction of PDLSCs, followed by the treatment of PDLSCs with proanthocyanidins. Lactylation eraser activator theophylline (Theo-24) was applied separately or simultaneously to test the relationship between lactylation levels and the osteogenic differentiation of PDLSCs after proanthocyanidin treatment. Western blot results showed that compared with the control group, proanthocyanidin treatment increased the expression of proteins related to the osteogenic differentiation of PDLSCs, accompanied by a significant increase in the level of lactylation, while the reduction in lactylation by Theo-24 significantly reduced the expression of proteins related to the osteogenic differentiation of PDLSCs, such as COL-1, ALP, RUNX2, and BMP2 (Figure 4A), and was statistically significant (Figure 4B). qRT-PCR results also showed the same trend, i.e., Theo-24 was able to inhibit the elevating effect of proanthocyanidins on the osteogenic differentiation genes of PDLSCs (Figure 4C). Compared with the proanthocyanidin-treated group, alkaline phosphatase and mineralized nodule formation in PDLSCs were significantly reduced after Theo-24 treatment, and Theo-24 was able to partially inhibit the pro-generative effects of proanthocyanidins on alkaline phosphatase and mineralized nodules in PDLSCs (Figure 4D) with statistical significance (Figure 4E). Immunofluorescence results showed that Theo-24 inhibited the enhancing effect of proanthocyanidins on the fluorescence intensity of Pan Kla and RUNX2 (Figure 4F), and the fluorescence intensity of Pan Kla and RUNX2 was correlated (Figure 4G). These results suggest that the osteogenic differentiation ability of proanthocyanidin-treated PDLSCs was significantly enhanced in the inflammatory state, possibly due to the up-regulation of lactylation. The inhibition of lactylation levels in PDLSCs would suppress their osteogenic differentiation ability.

In summary, the osteogenic differentiation of PDLSCs was significantly reduced in the LPS-induced inflammatory state, which was accompanied by a decrease in lactate production and lactylation levels, whereas proanthocyanidins were able to restore the suppressed osteogenic differentiation ability in the inflammatory state by restoring the lactylation levels of PDLSCs.

### 2.5. Proanthocyanidins Restore Lactylation in Inflammatory States and May Promote Osteogenesis in PDLSCs via the Wnt/β-Catenin Pathway

**Figure 5:**
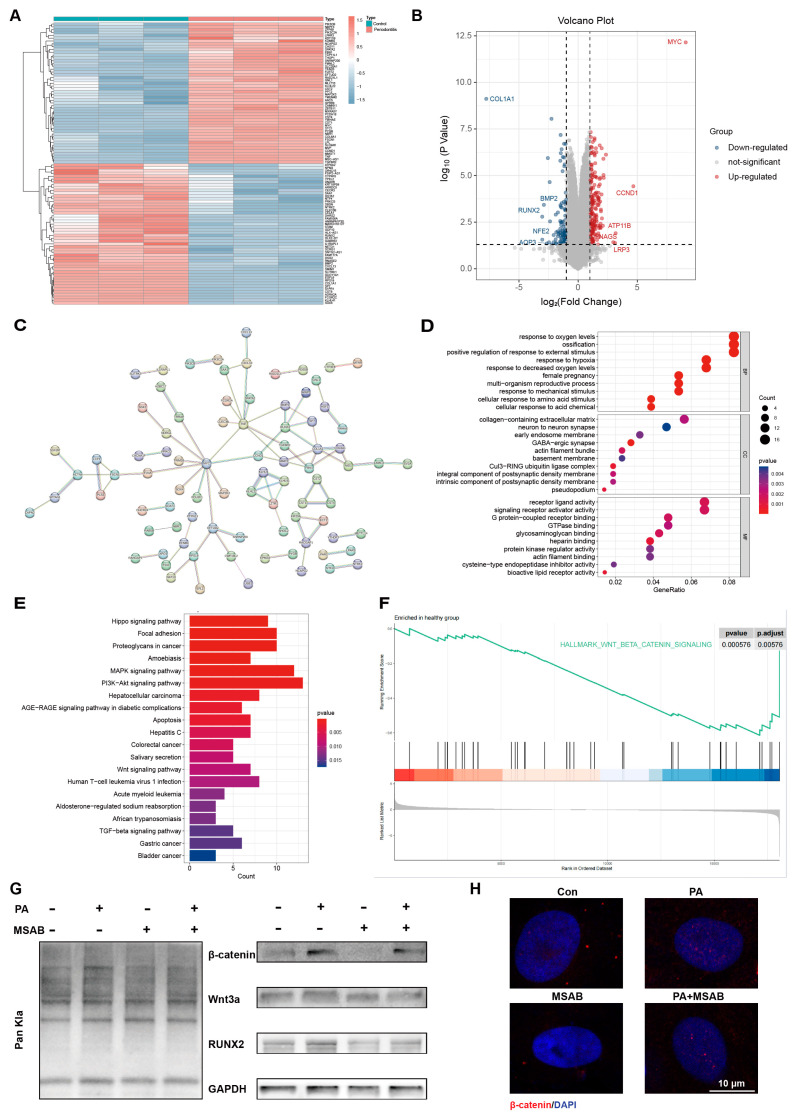
Restoration of lactylation by the proanthocyanidins-recovered osteogenesis of inflamed PDLSCs via the Wnt/β-catenin pathway. (**A**) Heatmap and (**B**) volcano plot showing differential genes between PDLSCs from normal and periodontitis tissues. (**C**) Interaction plot. (**D**) GO enrichment plots in PDLSCs from normal and periodontitis tissues. (**E**) KEGG analysis showing differential pathways in PDLSCs from normal and periodontitis tissues. (**F**) GSEA enrichment plots showing trends in the distribution of Wnt/β-catenin distribution trends in PDLSCs from normal tissues. (**G**) Western blotting showing the protein levels of Pan Kla, β-catenin, Wnt3a, and RUNX2 in PDLSCs after treatment with the conditions as shown. (**H**) Fluorescence confocal images showing the expression levels of β-catenin (red) in the cytoplasmic nuclei of PDLSCs after treatment with the conditions as shown. Nuclei were stained with DAPI (blue). PDLSCs: periodontal ligament stem cells; PA: proanthocyanidins (Control: PDLSCs from normal tissues, periodontitis: PDLSCs from periodontitis tissues, log2FC > 1, *p* < 0.01).

To explore the possible mechanism of proanthocyanidins’ pro-osteogenic effect on PDLSCs under an inflammatory state, we first analyzed the transcriptome sequencing results of PDLSCs extracted from periodontal membrane tissues of healthy and periodontitis groups from the database (GSE78074). The differential gene heatmap and volcano plot showed that compared with the healthy group, osteogenesis-related genes, such as *RUNX2*, *BMP2*, and *COL1,* in the periodontitis group decreased significantly (Figure 5A,B). The protein interaction network of differential genes showed that *RUNX2* was associated with both *BMP2* and *COL1* (Figure 5C). The Gene Ontology (GO) analysis showed that the differential genes were involved in the biological process of ossification (Figure 5D). The Kyoto Encyclopedia of Genes and Genomes (KEGG) analysis further showed that the Wnt/β-catenin signaling pathway, which is associated with osteogenesis, was affected by periodontitis and exhibited a significant decrease (Figure 5E). The Gene Set Enrichment Analysis (GSEA) showed that genes characteristic of the Wnt/β-catenin signaling pathway were enriched in the healthy group (Figure 5F). The expression level of *RUNX2*, as a downstream target gene of the Wnt/β-catenin pathway, was closely correlated with the Wnt/β-catenin pathway. Interestingly, our previous immunofluorescence correlation analysis showed that osteogenesis-associated RUNX2 had a high correlation with lactylation (Figure 2G and Figure 4G). Therefore, we sought to test whether lactylation promotes osteogenesis in PDLSCs through the activation of the Wnt/β-catenin pathway. MSAB, an inhibitor of the Wnt/β-catenin pathway, was used. The expression of the lactylation level, the Wnt/β-catenin pathway-related proteins β-catenin, Wnt3a, and the osteogenesis-related protein RUNX2 were examined, which showed that proanthocyanidins elevated the lactylation level, accompanied by the activation of the Wnt pathway and the elevation of the expression of RUNX2. The inhibition of the Wnt pathway by MSAB significantly reduced the pro-expression effect of proanthocyanidins on RUNX2 (Figure 5G). ALP and ARS results also showed that MSAB significantly attenuated the alkaline phosphatase and calcium nodule-promoting ability of proanthocyanidins on PDLSCs (Figure S2). Confocalization showed increased β-catenin entry into the nucleus and activation of the Wnt/β-catenin pathway after proanthocyanidins treatment of PDLSCs (Figure 5H). These results suggest that proanthocyanidins restoring the lactylation of PDLSCs in inflammatory states may enhance RUNX2 expression through the Wnt pathway, thereby promoting osteogenesis in PDLSCs.

## 3. Discussion

To date, periodontitis-related alveolar bone loss remains a difficult problem in the treatment of periodontitis [26]. An increasing number of studies have shown that epigenetic inheritance is involved in various aspects of osteogenesis. During osteogenesis, DNA methylation in the promoter region regulates ALP gene expression, and hypomethylation leads to the up-regulation of ALP genes. Conversely, promoter hypermethylation leads to decreased ALP expression in mature osteoblasts [27,28]. H3K4 hypermethylation of Runx2, ALP, OCN, and BSP genes mediates the differentiation of MSCs into the osteogenic lineage [29]. Lysine lactylation, a novel epigenetic modification, was found to promote the differentiation of bone marrow MSCs to osteoblasts [21], suggesting that lactate, as an important metabolite, plays an important role in osteogenesis, which links cellular metabolism and gene regulation. In this study, we used lactate production as an indicator of cellular metabolism (i.e., glycolysis) to dissect the intertwined association between lactate lactylation and osteogenesis and explored the change in the lactylation of PDLSCs in an inflammatory state and its relationship with osteogenesis for the first time. In our in vivo study, a histological assay found that both osteogenesis-related protein RUNX2 expression and lactylation level were down-regulated in periodontitis. Further, we used a lipopolysaccharide to induce inflammation and examined the lactylation levels of PDLSCs in the inflammatory state, and our results showed that the inflammatory state of PDLSCs was accompanied by a reduction in their osteogenic differentiation capacity and a decrease in lactylation level. After restoring lactylation using TSA, we found that the osteogenic capacity of PDLSCs was recovered, and the changes in both osteogenesis-related RUNX2 and lactated Pan Kla were significantly correlated. It was concluded that the reduced osteogenic differentiation capacity of PDLSCs in the inflammatory state was associated with the reduced lactylation level, and the restoration of the lactylation level could recover their osteogenic capacity.

The osteogenic potential of PDLSCs is important in repairing periodontitis-related alveolar bone loss [7,9,26,30,31,32]. Procyanidin B2 has been reported to restore the osteogenic capacity of human periodontal ligament fibroblasts in the inflammatory state [24]. Interestingly, we found that proanthocyanidins promoted osteogenic differentiation and enhanced lactylation levels in PDLSCs. After the inhibition of lactylation with lactylation eraser activator theophylline, the ability of proanthocyanidins to promote osteogenesis in PDLSCs was diminished. We, therefore, conclude that proanthocyanidins might recover the osteogenesis of PDLSCs in the inflammatory state by elevating the lactylation level of PDLSCs.

Previous findings suggest that proanthocyanidins may exert multiple effects on cells, including antioxidant [33,34,35], anti-inflammatory [36,37], and metabolic regulation [38,39]. Moreover, proanthocyanidins are reported to be able to influence the post-translational modifications of proteins. Proanthocyanin B2 can lead to the deacetylation of mitochondrial PKM2 and thereby attenuate pulmonary ischemia–reperfusion injury [40]. Deacetylation contributes to the formation of highly reactive tetrameric PKM2 and thus increases lactate production, which can directly influence lactylation levels [41]. However, the direct effects of proanthocyanidins on lactation have not been documented before. Here, we demonstrate that proanthocyanidins restore the osteogenic capacity of PDLSCs in inflammatory states. Proanthocyanidin treatment enhanced both the lactylation and expression of osteogenesis-related protein. The changes in osteogenesis-related RUNX2 and Pan Kla are significantly correlated. Osteoblast differentiation is highly modulated by cross-talk between genes, transcription factors, signaling pathways, and epigenetic mechanisms [42]. Epigenetic modifications alter chromatin structure and change the accessibility of genes to transcription and other regulatory factors. During a cell’s lifespan, these changes contribute largely to the up- and down-regulation of specific genes [43]. WNT family proteins are regarded as important signaling networks in both the maintenance and healing of craniofacial tissues, including periodontium [44]. In the current study, we found that the restoration of lysine lactylation by proanthocyanidins increased the expression level of β-catenin of PDLSCs, which represents the activation of the classical Wnt/β-catenin signaling pathway. In summary, proanthocyanidins recover the osteogenesis of PDLSCs under an inflammatory state by restoring lactylation, which is dependent on the activation of the Wnt classical signaling pathway. 

Proanthocyanidins have been reported to inhibit β-catenin expression levels in melanoma cells, which, in turn, inhibit tumor cell growth and promote apoptosis [45]. This seems to contradict our results of Wnt pathway activation after proanthocyanidin treatment. However, different cells do not respond to the drug in exactly the same way. Tumor cells have a typical Warburg effect [46,47], generating a large amount of ATP and lactate through aerobic glycolysis, which is reported to cause extremely high lactylation levels in tumor cells [17,48,49]. For example, compared to normal cells, the ocular melanoma cells have elevated levels of lactylation, thereby favoring tumor development, and the inhibition of lactylation levels can inhibit tumor development [18]. A similar phenomenon was also reported in tumors, such as hepatocellular carcinoma [50] and breast cancer [51,52]. 

**Figure 6:**
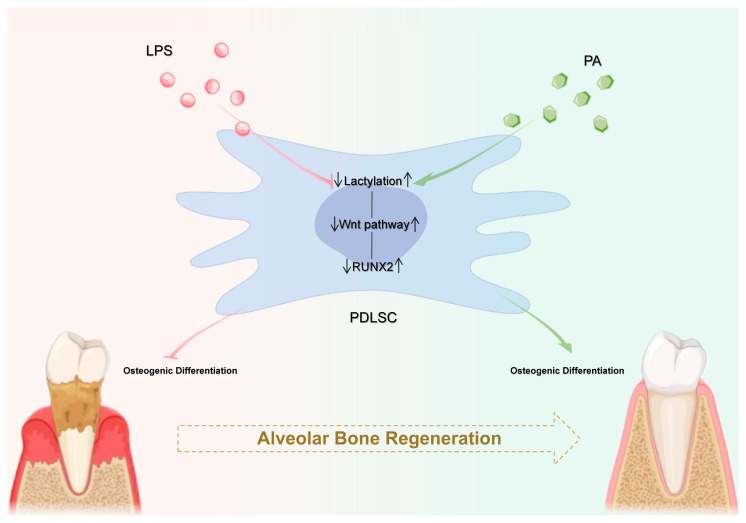
Schematic illustration. The decreased lactylation level of PDLSCs in an LPS-induced inflammatory state leads to the down-regulation of the Wnt/β-catenin pathway, which, in turn, reduces the expression of the Wnt downstream target gene RUNX2 and ultimately inhibits the osteogenic differentiation of PDLSCs. Proanthocyanidins enhance the osteogenic ability of PDLSCs by restoring the lactylation of PDLSCs, thereby up-regulating the Wnt/β-catenin pathway, increasing the expression of the downstream target gene RUNX2, and achieving the promotion of alveolar bone regeneration. PDLSCs: periodontal ligament stem cells; LPS: lipopolysaccharide; PA: proanthocyanidins.

In summary, we found impaired osteogenesis of PDLSCs in the inflammatory state was associated with the decreased lysine lactylation and restoration of the lactylation by the proanthocyanidins-recovered osteogenesis of inflamed PDLSCs through the canonical Wnt/β-catenin signaling pathway (Figure 6). Future studies could identify specific sites of lysine lactylation by liquid chromatography–mass spectrometry followed by chromatin immunoprecipitation to further determine the relationship between lactylation and the Wnt/β-catenin signaling pathway. 

Lactylation occurs on lysine residues of proteins, including histone lactylation and non-histone lactylation. The limitation of this study is that the specific lysine sites where lactylation occurs were not identified, and in addition, other possible related signaling pathways may require further investigation.

## 4. Materials and Methods

### 4.1. Animal Experiments

Animal experiments were conducted in accordance with the approved protocol of the Animal Experimentation Committee of Tongji Hospital, Tongji Medical College, Huazhong University of Science and Technology (Approval No. TJH-202201031). Six 6-week-old male Sprague Dawley (SD) rats were procured from the Experimental Animal Center of Tongji Medical College, Huazhong University of Science and Technology, and they were all housed under specific pathogen-free conditions. The rats were randomly divided into two groups (n = 3): the blank control group (healthy mice without periodontitis, NC) and the periodontitis group (periodontitis). In the periodontitis group, the SD rats were anesthetized with 1% sodium pentobarbital (40 mg/kg body weight), and then 0.20 mm orthodontic ligature wires with 3-0 wire were placed between the maxillary first and second molars. An inoculating loop was used to apply a suspension of *Porphyromonas gingivalis* (*P. gingivalis*) at a concentration of 1 × 10^8^ CFU/mL to the gingival sulcus and the ligation wire. The wire was inoculated every three days while checking for the presence of the ligation wire until the end of the six-week period. The rats were then euthanized with 1% sodium pentobarbital (40 mg/kg body weight) after being anesthetized. A portion of the maxilla, including maxillary molars and alveolar bone, was obtained and fixed in 4% paraformaldehyde (Servicebio, Wuhan, China) for two days. The samples were decalcified in 12% EDTA (Sigma-Aldrich, St. Louis, MO, USA) for 30 days. The samples were embedded in paraffin wax (Leica, Wetzlar, Germany) and serially sectioned along the medial–distal direction of the buccal roots of the first and second molars, obtaining 4 μm thick sections (Leica). Finally, Hematoxylin and eosin (H&E) stains were used to examine histologic changes in the periodontal tissue of the tissue sections. RUNX2 (ABclonal, Wuhan, China) and Pan-Kla (PTM Bio, Hangzhou, China) immunohistochemistry (IHC) assays were used to detect the lactylation levels.

### 4.2. Isolation and Culture of PDLSCs and Relative Reagents

Healthy premolars were obtained with the consent of 20 patients (aged 14–20 years) who needed to have their premolars extracted for orthodontic treatment. The protocol was approved by the Medical Ethics Committee of Tongji Hospital, Huazhong University of Science and Technology (Protocol No. TJ-IRB20221303). The exclusion criteria were (i) significant caries and (ii) severe periodontal disease. The extracted premolars were placed in MEM alpha-modified medium (α-MEM; Boster, Wuhan, China) with 5% antibiotics (100 U/mL penicillin, 10 mg/mL streptomycin; Boster, Wuhan, China). The cells were rinsed five times with phosphate buffer solution (PBS; Boster, Wuhan, China) containing 5% antibiotics, and the periodontal membrane tissue was scraped from the middle of the root surface with a sterile blade, and then the primary cells were cultured in α-MEM medium containing 20% fetal bovine serum (FBS; Gibco, ThermoFisher Scientific, Waltham, MA, USA) and 1% antibiotics. When the primary cells grew to 80–90% confluence, the cells were digested with trypsin and centrifuged, resuspended in α-MEM medium containing 10% fetal bovine serum and 1% antibiotics as the first generation of cells (P1), and incubated in 5% CO_2_ at 37 °C, with fresh medium changes every 2–3 days. Cells from passages 2 to 4 were used for subsequent experiments.

*P. gingivalis*-derived LPS was purchased from Invivogen (Toulouse, France). Grape seed oligomeric proanthocyanidins were purchased from Sigma-Aldrich (St. Louis, MO, USA). A lactylation eraser inhibitor, Trichostatin A (TSA), a lactylation eraser activator, theophylline (Theo-24), and an inhibitor of the Wnt/β-catenin pathway, MSAB, were purchased from MedChemExpress (Monmouth Jumction, NJ, USA).

### 4.3. Immunohistochemistry

The obtained samples were immediately fixed in 4% paraformaldehyde, decalcified with 10% EDTA, and embedded in paraffin blocks. Tissue sections with a thickness of 5 µm were prepared using a slicer. Paraffin-embedded tissue sections were dewaxed in xylene and rehydrated by graded alcohol with water. Endogenous peroxidase was blocked using 3% H_2_O_2_ for 15 min. Antigen repair using 0.3% trypsin was performed for 15 min. Sections were closed with 10% serum for 30 min. The slides were incubated with anti-RUNX2 and anti-Kla primary antibodies, respectively, at 4 °C overnight, and then the slides were rewarmed at room temperature and incubated with goat anti-rabbit secondary antibodies applied for one hour at room temperature. The sections were then incubated in a streptavidin–biotin complex (SABC; Boster, Wuhan, China) for 30 min. Diaminobenzidine (DAB; ThermoFisher Scientific, Waltham, MA, USA) solution was applied for 2–5 min, and the development of color reaction was monitored by microscopy. Slides were re-stained with hematoxylin, dehydrated, cleaned, and then fixed. The slides were observed under a light microscope (BX-51, Olympus, Tokyo, Japan) and imaged. 

### 4.4. Lactate Concentration Detection

A total of 5 × 10^6^ cells were resuspended using 1 mL of PBS, and the cells were broken by ultrasonic waves in an ice bath (power of 300 volts, ultrasonic waves for 3 s, interval of 7 s, total time of 3 min). Centrifugation was carried out at 4 °C, 12,000× *g* for 10 min, and the supernatant was harvested. A total of 1 mL of enzyme working solution and 0.2 mL of color-developing solution (Jiancheng, Nanjing, China) were added to 0.02 mL of the supernatant and reacted for 10 min at 37 °C in a water bath. Then, 2 mL of terminating solution (Jiancheng, Nanjing, China) was added to each tube and mixed well. The enzyme-labeled instrument was used to determine the absorbance value of each tube at a wavelength of 530 nm. The content of lactic acid was according to the standard curve.

### 4.5. qRT-PCR Analysis

The mRNA levels of runt-related transcription factor 2 (*RUNX2*), alkaline phosphatase (*ALP*), collagen type I (*COL-1*), and bone morphogenetic protein 2 (*BMP2*) were assessed by qRT-PCR. The cells were first washed three times with cold PBS, and total RNA was isolated using the RNA Extraction Kit (Accurate Biology, AG21017). cDNA was synthesized using the Total Transcriptome CDNA Synthesis Kit (Accurate Biology, AG11707). cDNAs were normalized to *GAPDH*, respectively. Primers were designed with Primer Premier 5.0 software and listed in Table 1. A comparison of the 2^−ΔΔCt^ method was used to quantify the relative expression levels of mRNAs.

### 4.6. Western Blot Analysis

Cells were washed three times with cold PBS, and then total cellular proteins were lysed with a protein extraction kit (ThermoFisher Scientific, Waltham, MA, USA). The lysate was centrifuged at 12,000× *g* for 15 min, and the supernatant was collected to determine the protein concentration using the BCA Protein Assay Kit (Epizyme, Shanghai, China). The samples and BSA markers were loaded onto a 10% SDS-PAGE gel and transferred to a PVDF membrane, which was closed in 5% skimmed milk for 1 h. Primary antibodies were incubated at 4 °C overnight. Subsequently, the membrane was washed three times with triple-buffered saline (TBST) containing Tween 20 for 10 min each, incubated with the secondary antibody for 1 h at room temperature, and then washed three times with TBST for 15 min each. The bands were visualized using chemiluminescent reagents. Protein expression was quantified using Image J software (at http://imagej.nih.gov/, NIH, Bethesda, MD, USA). The expression levels of each protein were normalized to GAPDH for statistical analysis.

Primary antibodies are as follows: rabbit ant-IL-1β (1:1000, Cell Signaling Technology, Danvers, MA, USA); rabbit anti-coll I antibody (1:1000, ABclonal, Wuhan, China); rabbit anti-ALP antibody (1:1000, ABclonal, Wuhan, China); rabbit anti-RUNX2 antibody (1:1000, ABclonal, Wuhan, China); rabbit anti-BMP2 antibody (1:1000, ABclonal, Wuhan, China); and rabbit anti-Pan Kla antibody (1:1000, PTM Bio, Hangzhou, China). Secondary antibodies are as follows: anti-rabbit IgG, HRP-linked antibody (1:1000, ABclonal, Wuhan, China), and anti-mouse IgG, HRP-linked antibody (1:1000, ABclonal, Wuhan, China).

### 4.7. Alkaline Phosphatase (ALP) Staining

Cells were seeded in 6-well plates at a density of 2 × 10^5^ cells/well in α-MEM medium supplemented with FBS (10%), dexamethasone (10 nM, Sigma-Aldrich, St. Louis, MO, USA), β-glycerophosphate (10 mM, Sigma-Aldrich, St. Louis, MO, USA), and L-ascorbic acid (50 µM, Sigma-Aldrich, St. Louis, MO, USA), which were used as osteogenic induction media. LPS, proanthocyanidins, TSA, theophylline (Theo-24), and MSAB were added according to the experimental groups and cultured in an osteogenic induction medium. The medium was changed every 3 days until 7 days of culture, and the cells were fixed in 4% paraformaldehyde for 10 min and then stained for ALP using the NBT/BCIP staining kit, according to the manufacturer’s instructions. Quantification was performed using Image J software (NIH, Bethesda, Maryland, USA).

### 4.8. Alizarin Red S(ARS) Staining

Cells were seeded in 6-well plates at a density of 2 × 10^5^ cells/well and cultured with LPS, proanthocyanidins, TSA, theophylline, and MSAB, which were added according to the experimental groups and cultured in an osteogenic induction medium for 21 days, rinsed with PBS three times, fixed in 4% paraformaldehyde solution for 20 min, and stained with 2% alizarin red S solution for 10 min. Photographs of staining results were taken under an inverted microscope and scanned with a scanner. Quantification was performed using Image J software (NIH, Bethesda, Maryland, USA).

### 4.9. Immunofluorescence

Cells cultured on coverslips were fixed with 4% paraformaldehyde for 20 min at room temperature and then rinsed with cold PBS three times. The membranes were broken with 0.5% Triton X-100 (Servicebio, Wuhan, China) solution for 5 min and then washed with cold PBS 3 times. Cells were blocked with blocking solution for 30 min and incubated overnight at 4 °C with the indicated primary antibodies. After 3 washes with cold PBS, the cells were incubated with fluorescein isothiocyanate or rhodamine-conjugated secondary antibody for 3 h at room temperature. Cells were counterstained with 100 ng/mL 4′,6-diamidino-2-phenylindole (DAPI, Servicebio, Wuhan, China) for 5 min to visualize the nuclear DNA. Coverslips were mounted on glass slides with an anti-quenching solution and viewed under an OLYMPUS IX71 inverted microscope (Olympus, Tokyo, Japan).

### 4.10. Statistical Analysis

All data were collected and expressed as the mean ± standard deviation (SD) of three independent experiments. Tests were analyzed using GraphPad Prism software (MacKiev Software, version 6, Boston, MA, USA), and differences among groups were analyzed using the one-way ANOVA followed by a Tukey HSD comparison test after validating the normality and homoscedasticity assumptions of the data set. If either assumption was violated, the data set was transformed logarithmically prior to the application of the aforementioned parametric statistical analysis methods. A statistical probability of *p* < 0.05 was considered significant.

## 5. Conclusions

In this study, we examined for the first time the lactylation level of PDLSCs under an LPS-induced inflammatory state and found that the lactylation of PDLSCs was reduced, providing a new reference for the mechanism of the impaired osteogenic differentiation of PDLSCs in the inflammatory microenvironment. We also found that proanthocyanidins were able to promote PDLSC osteogenesis by restoring lactylation, providing a new basis for the specific mechanism by which proanthocyanidins promote the osteogenic differentiation of PDLSCs in the inflammatory state. Finally, we conducted a preliminary exploration of the possible mechanisms of lactylation to promote osteogenesis and found that elevated lactylation levels are closely related to the activation of the Wnt pathway. Proanthocyanidins may activate the Wnt pathway by elevating lactylation levels in the inflammatory state, which, in turn, promotes the osteogenesis of PDLSCs. We anticipate that this study will provide a valuable reference for the mechanism exploration and clinical application of proanthocyanidins and PDLSCs in periodontal tissue regenerative engineering.

## Figures and Tables

**Table 1:** Primer sequences.

Genes	Forward Primer Sequences (5′-3′)	Reverse Primer Sequences (5′-3′)
*COL1A1*	TAGGGTCTAGCATGTTCAGCTTTG	CGTTCTGTACGCAGGTGATTG
*ALPL*	GGACCATTCCCACGTCTTCA	CAGGCCCATTGCCATACA
*RUNX2*	CACTGGCGCTGCAACAAGA	CATTCCGGAGCTCAGCAGATAA
*BMP2*	CCACCATGAAGAATCTTTGGA	GAGTTGGCTGTTGCAGGTTT
*GAPDH*	GGTGAAGGTCGGAGTCAACG	CAAAGTTGTCATGGATGHACC

## Data Availability

The data that support the findings of this study are available from the corresponding author upon reasonable request.

## Supplementary Materials

The following supporting information can be downloaded at https://www.mdpi.com/article/10.3390/ijms25052947/s1.
