# Supplementary material for: Proanthocyanidins Ameliorate LPS-Inhibited Osteogenesis of PDLSCs by Restoring Lysine Lactylation
Source: Int J Mol Sci. 2024 Mar 3;25(5):2947. doi: 10.3390/ijms25052947 (PMC10931601; doi:10.3390/ijms25052947)
Supplement: Supplementary file 1 [file ijms-25-02947-s001.zip › ijms-2875273-supplementary.pdf]

Proanthocyanidins Ameliorate LPS-Inhibited Osteogenesis of PDLSCs by Restoring Lysine Lactylation

Yaxin Wu [1,2], Xiangyao Wang [1,2], Yuxiao Zhang [1,2], Zhihao Wen [1,2], Yuanyuan Li [1,2], Kehan Zhang [1,2], Nuerlan Gosar [1,2], Qilin Li [1,2], Jing Mao [1,2,*] and Shiqiang Gong [1,2,*]

Affiliations

1 Department of Stomatology, Tongji Hospital, Tongji Medical College, Huazhong University of Science and Technology, Wuhan 430030, China

2 School of Stomatology, Tongji Medical College, Huazhong University of Science and Technology, Wuhan 430033, China

Supplementary materials

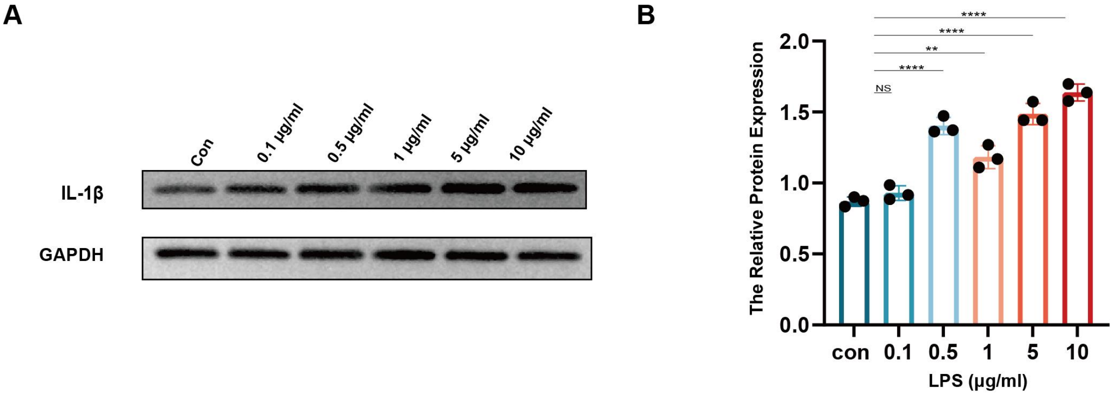

Figure S1. P. gingivalis-derived LPS construct the inflammation model of PDLSCs .

(**A**) Western blotting showing protein levels of IL-1β. (**B**) Quantitative analysis of IL-1β protein levels. ** $p < 0.01$, **** $p < 0.0001$, NS indicates no statistical difference.

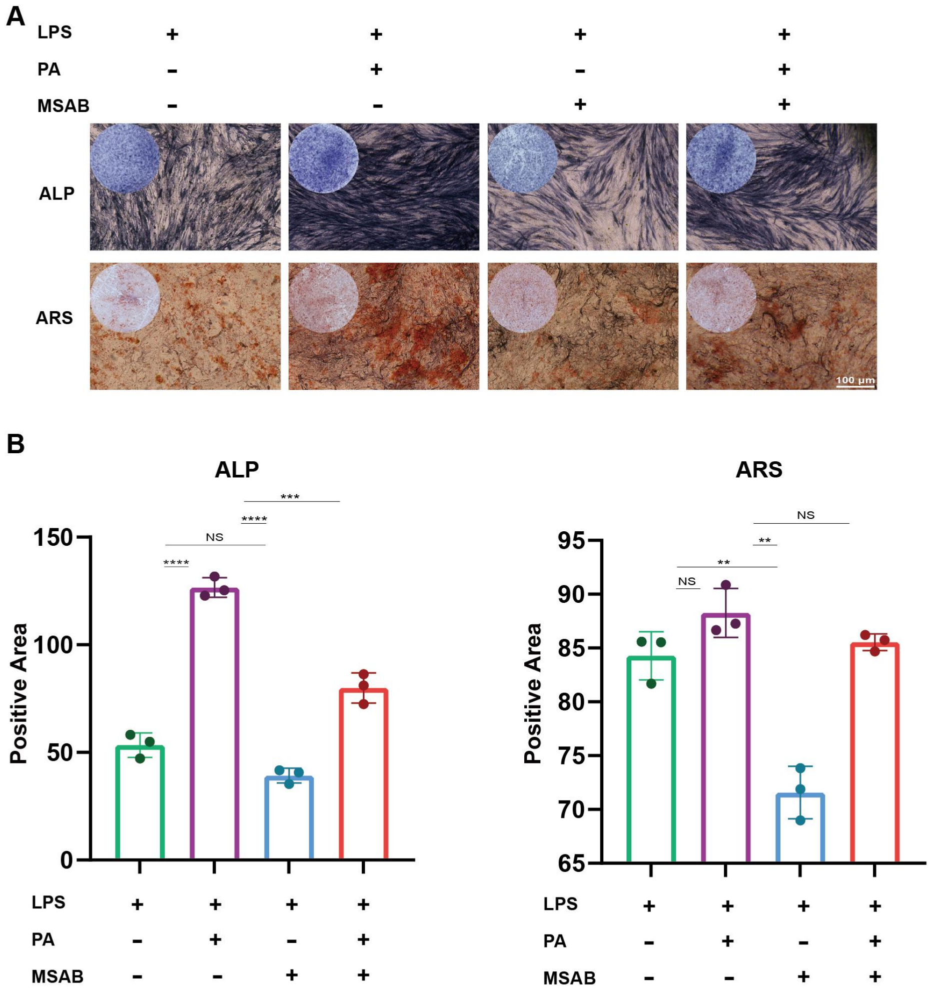

Figure S2. MSAB attenuate the alkaline phosphatase and calcium nodule-promoting ability of proanthocyanidins on PDLSCs.

(**A**) ALP staining at 7 days and ARS staining at 21 days of osteogenic induction in PDLSCs. (**B**) Quantitative analysis of ALP staining and ARS staining. ** $p < 0.01$, *** $p < 0.001$, **** $p < 0.0001$, NS indicates no statistical difference
